# Transcriptional Patterns of Biogeochemically Relevant Marker Genes by Temperate Marine Bacteria

**DOI:** 10.3389/fmicb.2020.00465

**Published:** 2020-03-20

**Authors:** Laura Alonso-Sáez, Xosé Anxelu G. Morán, José M. González

**Affiliations:** ^1^Marine Research Division, AZTI, Sukarrieta, Spain; ^2^Centro Oceanográfico de Gijón/Xixón, Instituto Español de Oceanografía (IEO), Gijón/Xixón, Spain; ^3^Biological and Environmental Sciences and Engineering Division, Red Sea Research Center, King Abdullah University of Science and Technology (KAUST), Thuwal, Saudi Arabia; ^4^Department of Microbiology, University of La Laguna, La Laguna, Spain

**Keywords:** gene expression, metatranscriptomics, coastal, bacterioplankton, functional marker genes

## Abstract

Environmental microbial gene expression patterns remain largely unexplored, particularly at interannual time scales. We analyzed the variability in the expression of marker genes involved in ecologically relevant biogeochemical processes at a temperate Atlantic site over two consecutive years. Most of *nifH* transcripts, involved in nitrogen (N) fixation, were affiliated with the symbiotic cyanobacterium *Candidatus* Atelocyanobacterium thalassa, suggesting a key role as N providers in this system. The expression of *nifH* and *amoA* (i.e., marker for ammonia oxidation) showed consistent maxima in summer and autumn, respectively, suggesting a temporal succession of these important N cycling processes. The patterns of expression of genes related to the oxidation of carbon monoxide (*coxL*) and reduced sulfur (*soxB*) were different from that of *amoA*, indicating alternate timings for these energy conservation strategies. We detected expression of alkaline phosphatases, induced under phosphorus limitation, in agreement with the reported co-limitation by this nutrient at the study site. In contrast, low-affinity phosphate membrane transporters (*pit)* typically expressed under phosphorus luxury conditions, were mainly detected in post-bloom conditions. *Rhodobacteraceae* dominated the expression of *soxB, coxL* and ureases, while *Pelagibacteraceae* dominated the expression of proteorhodopsins. *Bacteroidetes* and *Gammaproteobacteria* were major contributors to the uptake of inorganic nutrients (*pit* and *amt* transporters). Yet, in autumn, *Thauma*- and *Euryarchaeota* unexpectedly contributed importantly to the uptake of ammonia and phosphate, respectively. We provide new hints on the active players and potential dynamics of ecologically relevant functions *in situ*, highlighting the potential of metatranscriptomics to provide significant input to future *omics*-driven marine ecosystem assessment.

## Introduction

The analysis of marine protein-coding genes has undergone rapid expansion following the development of *meta-omics* technologies and its application to oceanic environments, from local to large-scale ocean surveys (Gilbert and Dupont, 2011; Moran et al., 2013; Salazar et al., 2019). The study of this vast repertoire of microbial functional genes is providing new perspectives on the ocean biogeochemistry, including the discovery of novel enzymes, pathways, and key microbial players (Zehr and Kudela, 2011; Ferrera et al., 2015). In this regard, the identification of marker genes, highly specific for some metabolic functions, has been key to address the abundance, diversity and geographic extent of microbes involved in biogeochemically relevant processes, such as nitrification (*amoA* genes, Francis et al., 2005; Sintes et al., 2013), nitrogen fixation (*nifH* genes; Zehr et al., 2003), or carbon monoxide oxidation (*cox* genes; King and Weber, 2007).

As compared to the oceanic distribution of these functional marker genes (as targeted by metagenomics), our knowledge on their gene expression patterns has grown at a slower pace. In one the first applications of metatranscriptomics to aquatic environments, some biogeochemically relevant processes such as bacterial assimilation of C_1_ compounds and the oxidation of sulfur (S) compounds were found to be active at a coastal Atlantic salt marsh (Poretsky et al., 2005). Related with C_1_ metabolism, carbon monoxide (CO) is an important greenhouse gas, which in marine systems is mainly produced by photochemical degradation of organic matter. Thus, the uptake of this compound by marine bacteria can substantially reduce its oceanic emissions to the atmosphere (King and Weber, 2007). Even if the marker genes for CO oxidation (*coxL*) are abundant in the environment (Cordero et al., 2019), reports of their expression in marine waters are still scarce (Georges et al., 2014). Also linked to climate, dimethylsulfoniopropionate (DMSP) is a source of C and reduced S for marine microorganisms, and the precursor of the climate-cooling gas DMS (Yoch, 2002). While the production of DMS from DMSP was first described in Rhodobacteraceae, other key players in this process, such as some members of Gammaproteobacteria, have been identified by experimental metatranscriptomics (Vila-Costa et al., 2010).

In the context of the nitrogen (N) cycle, studies targeting the functional marker genes *amoA* (for ammonia oxidation) and *nifH* (for nitrogen fixation) have drastically increased our knowledge on the magnitude of these two processes in the ocean. In the former case, the sequencing of some of the first marine metagenomes retrieved *amo* sequences of archaeal origin (Venter et al., 2004), challenging the previous assumption that this function was exclusively carried out by a few members of *Gamma-* and *Betaproteobacteria* (Ward et al., 2007). Subsequent studies have found that Archaea actually dominate the oxidation of ammonia in the ocean, expanding the geographical distribution of this process to the extensive mesopelagic and bathypelagic realms (Wuchter et al., 2006; Sintes et al., 2013, 2016). In the second case, biological N fixation, which reduces atmospheric N_2_ to biologically available ammonium, supplies this essential nutrient to aquatic ecosystems. First thought to be dominated by the filamentous bloom-forming cyanobacteria *Trichodesmium*, this process is now known to be mediated mainly by widespread unicellular N_2_ fixers in the open ocean (such as *Candidatus Atelocyanobacterium thalassa* and *Crocosphaera watsonii*, Zehr, 2011). Additionally, there is evidence of heterotrophic bacteria involved in this process (Bombar et al., 2016). These findings have changed the size class of the dominant diazotrophs in the ocean, with implications in the fate of fixed N in marine food webs. Determining the spatial coverage and temporal activity of these widespread, abundant unicellular diazotrophs would be a key step toward filling the large gaps in our oceanic global N_2_ fixation estimates.

In general, marine metatranscriptomic studies have helped uncover the key microbial players involved in different biogeochemical processes, and where in the ocean they become active. Yet, identifying the temporal patterns in the expression of globally relevant functional genes remains one of the main gaps in knowledge (Salazar et al., 2019). While there are evidences of diel oscillations in marine microbial expression patterns (Poretsky et al., 2009b; Ottesen et al., 2014; Aylward et al., 2015), few studies have addressed gene expression patterns at longer time-scales (e.g., monthly, inter-annually; but see Gifford et al., 2014; Hollibaugh et al., 2014). Here, we carried out an analysis of a metatranscriptomic dataset obtained over two consecutive years in surface waters at a temperate mid-shelf station in the southern Bay of Biscay (NE Atlantic). Our aims were (i) to identify key biogeochemical pathways that were active at the study site by following the expression dynamics of functional marker genes, (ii) to analyze the composition of transcriptionally active taxa involved in these functions, and (iii) to understand the variability in their expression levels under environmental conditions ranging from oligotrophic to mesotrophic, as typically found in temperate waters over the seasonal cycle.

## Materials and Methods

### Sample Collection

Eight metatranscriptomic samples were collected in spring (April and/or May), summer (July) and autumn (November) over two consecutive years at the station Radiales E2 Gijón/Xixón (43.67°N, 5.58°W) in the Southern Bay of Biscay. For consistency with previous studies on the same dataset (e.g., Alonso-Sáez et al., 2015, 2018), a sample collected in early May in 2012 (2nd May) has been designated “April 2012,” to distinguish it from a second sample taken in late May (23rd May, see Supplementary Table S1). Ancillary variables (temperature, salinity, chlorophyll *a*, bacterial cell abundance and heterotrophic prokaryotic production) were measured as explained in Arandia-Gorostidi et al. (2017). Samples for RNA analysis (from 4.5 to 11 L) were collected from a depth of 5 m and immediately filtered using 3-μm pore-size polycarbonate pre-filters and 0.22-μm pore-size polycarbonate filters (GTTP, Millipore). The 0.22-μm filters were placed in Whirl-Packs containing 2 mL of RLT buffer (Qiagen, Valencia, CA), flash frozen in liquid nitrogen, and stored at −80°C. Time from sample collection to flash freezing of the filters ranged between 15 and 20 min. Samples were taken around midday (1–3 h after noon), to minimize biases in the interpretation of the results due to differences in diel transcription patterns (Poretsky et al., 2009b).

### RNA Processing

RNA was extracted as previously detailed (Poretsky et al., 2009a). Briefly, filters were shattered with a mallet, vortexed in falcon tubes containing Power Soil beads (Mobio), and the lysate was mixed with 70% ethanol (1:1 volume). RNA extractions were carried out with the RNeasy Mini Kit (Qiagen). RNA was treated with Turbo DNase (Ambion) and the ribosomal RNA was depleted using the mRNA-only isolation kit (Epicenter), the MicrobeExpress, and MicrobeEnrich kits (Ambion). The enriched mRNAs were linearly amplified using the Message Amp II-Bacteria kit (Ambion), reverse transcribed to double-stranded complementary DNA (cDNA) with the Universal Riboclone cDNA synthesis system (Promega) and purified with the QIAQuick PCR purification kit (Qiagen). The eight cDNA samples were subjected to single-end sequencing in an Illumina MiSeq run.

### Bioinformatics Analysis

After an initial quality trimming of the reads, ribosomal RNAs (rRNAs) were removed from the dataset after identifying them by a BLASTn search using a SILVA reference database. The sequence of phiX174, used as a control in Illumina platforms, was also removed prior to further analyses. The remaining non-rRNA sequences were annotated using two different workflows. First, for a preliminary identification of functional marker protein-coding genes and top-hit taxonomic bins, all putative mRNAs were queried (BLASTx, bitscore cutoff ≥40, Altschul et al., 1990) against the National Center for Biotechnology Information’s (NCBI) RefSeq database (version 63, January 2014), which includes proteins from viruses and all three domains of life. The annotations of the BLASTx top-hits were screened by text-based queries to search for specific functional genes following Gifford et al. (2013). Next, a subset of mRNAs representing an even coverage among samples (201510 sequences each) were queried by BLASTx (bitscore cutoff ≥40) against a custom RefSeq database where peptides of interest were reannotated using hidden Markov models run with HMMER3 (Eddy, 2008). The list of genes selected fulfilled two criteria: (i) high specificity for the corresponding function of interest (to avoid targeting genes with ambiguous functions or potentially involved in several metabolic processes) and, (ii) being represented by a single protein family easily recognizable by bioinformatic methods. Based on this selection, the protein families TIGR01287, TIGR01792, TIGR04486, TIGR01115, and TIGR00842 were used to search NifH, UreC, SoxB, PufM, and BCCT, respectively. PF05787, PF09423, and PF01384, were used for PhoX, PhoD, and Pit transporter searches, respectively. Finally, PF01315, PF00909, PF12942, and PF01036 were used for CoxL, amt transporters, archaeal AmoA and proteorhodopsin (PR) searches, respectively. TIGR03080 was used to search for bacterial AmoA, but no hits were found in our metatranscriptomes. A PFAM or TIGRFAM hit was considered valid if its score was equal to or bigger than the recommended gathering score for the hidden Markov model. The label of the peptides in the RefSeq sequence file was modified to accommodate the new annotation based on hits to protein families. In the case of CoxL, we additionally looked for the signature sequence “AYxCSFR” at the active site of the enzyme, a motif present only in Form I Carbon Monoxide Dehydrogenases (CODH). Thus, CODH Form II enzymes, which may not be primarily involved in CO oxidation (King and Weber, 2007), were not targeted in the analyses. In the case of the Pit and PR, a preliminary search against PATRIC protein families (Wattam et al., 2013) identified a substantial number of hits of euryarchaeal origin. Thus, euryarchaeal peptides were incorporated to the protein database as they were absent from RefSeq. The microbial taxa involved in each marker gene expression were identified using MEtaGenome Analyzer (MEGAN, Huson et al., 2007), to obtain consensus taxonomic assignments.

## Results and Discussion

**FIGURE 1:**
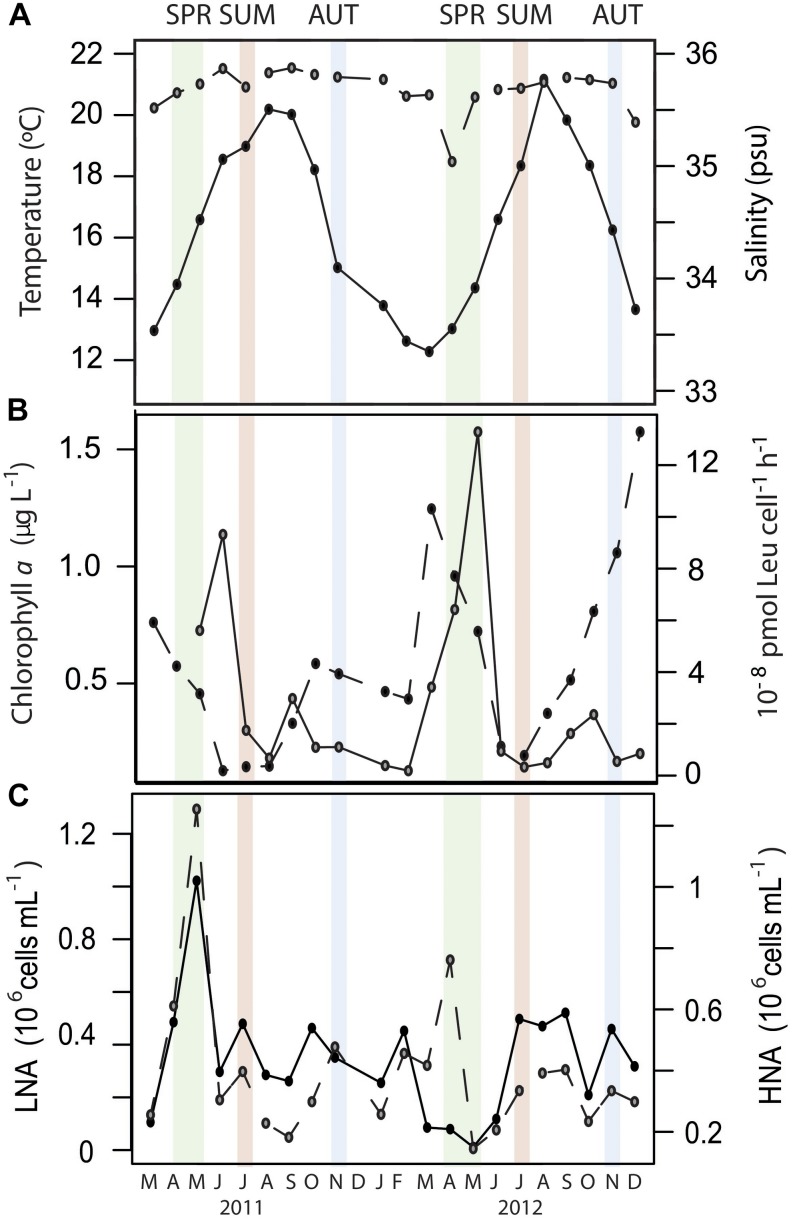
Monthly data at the surface of the study station (E2-Gijón/Xixón) from March 2011 until December 2012 for **(A)** surface temperature (closed circles) and salinity (open circles); **(B)** Chlorophyll *a* concentration (closed circles) and cell-specific bacterial heterotrophic production (open circles); **(C)** Abundance of low- nucleic acid (LNA, closed circles) and high-nucleic acid (HNA, open circles) cells. Months when samples for metatranscriptomic analyses were collected appear highlighted in colors (green for spring, red for summer and blue for autumn).

The temperate station of study (E2-Gijón/Xixón, 100 m maximum depth) is a long-term monitoring site (Morán et al., 2015) located ca. 13.5 km off the Spanish coast. This site is characterized by a late winter/early spring phytoplankton bloom, followed by summer thermal stratification and autumn re-mixing of the water column, like most of the northern Iberian Peninsula continental shelf (Bode et al., 1996; Morán et al., 2015). Samples for mRNA analysis were collected at different seasonal periods over two consecutive years, with the aim of covering a wide range of oceanographic conditions, from oligotrophic (summer stratification) to mesotrophic (mixing periods, Figure 1). Summer and autumn samples differed substantially in temperature and chlorophyll *a* concentration over the 2 years but shared relatively low levels of cell-specific heterotrophic bacterial production (Figure 1). Bacterial production was maximum in spring, concomitant with a declining trend in chlorophyll *a* concentration, characteristic of post-bloom scenarios (Figure 1). However, the two spring periods analyzed showed important differences in terms of bacterial abundance and cytometric profiles. An unusually low abundance of low-nucleic-acid cells *in situ* was found in May 2012 (Figure 1), reaching minimum levels in the first 10-years of the time-series at the study site (Morán et al., 2015). This change was also reflected in the taxonomic composition of bacterial communities in spring 2012, clearly differing from the two previous spring periods (Alonso-Sáez et al., 2015). This suggests that the spring samples collected in 2012 were rather atypical in environmental and/or biotic conditions, which resulted in a large year-to-year variation in the expression profiles in that season. In this line, the relative contributions of the 50 top-hit taxonomic bins to the transcript pool were highly correlated in samples taken in autumn or summer, but not in spring (Supplementary Figure S1).

From the 4.26 million reads identified as non-rRNA transcripts in the metatranscriptomes, 61% had significant hits in the RefSeq protein database (Supplementary Table S1). In a preliminary BLASTx search, several genes involved in ecologically relevant metabolic pathways (Moran, 2008) were detected at various expression levels in our dataset (Supplementary Table S2). We subsequently focused on a reduced set of well-studied marker genes, highly specific for environmentally relevant functions (Ferrera et al., 2015), and represented by single protein families, which would allow a robust method of detection (following Palovaara et al., 2014). These marker genes belonged to six main categories: photoheterotrophy (PR, *pufM*), oxidation of inorganic compounds (*coxL*, *amoA*, and *soxB* for carbon monoxide, ammonia and sulfide/thiosulfate oxidation, respectively), nitrogen acquisition (*nifH*, *ureC*, and *amt*, for nitrogenases, ureases and ammonia membrane transporters, respectively), phosphate acquisition (the extracellular alkaline phosphatases *phoX* and *phoD*, and the low-affinity phosphate membrane transporter *pit*) and reduced S acquisition (the Betaine/Carnithine/Choline BCCT family transporter involved in DMSP incorporation, Sun et al., 2011).

**FIGURE 2:**
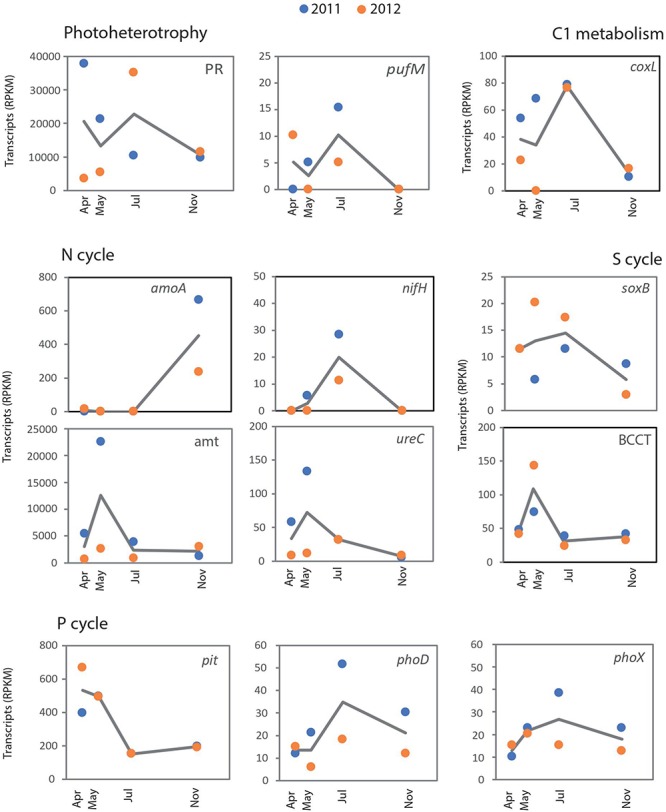
Temporal dynamics in expression patterns of key marker genes for photoheterotrophy (proteorhodopsins and aerobic anoxygenic photosynthesis), C_1_ metabolism (oxidation of carbon monoxide), N cycle (ammonia monooxygenase, nitrogenase, ammonia transporter and urease), S cycle (sulfur oxidation and DMSP uptake), and P cycle (low-affinity phosphate transporter and extracellular phosphatases) over two consecutive years. The marker genes analyzed for each case appear in each individual plot. For each marker gene, the normalized abundance of transcripts has been calculated as Reads per Kilobase Million (RPKM).

**FIGURE 3:**
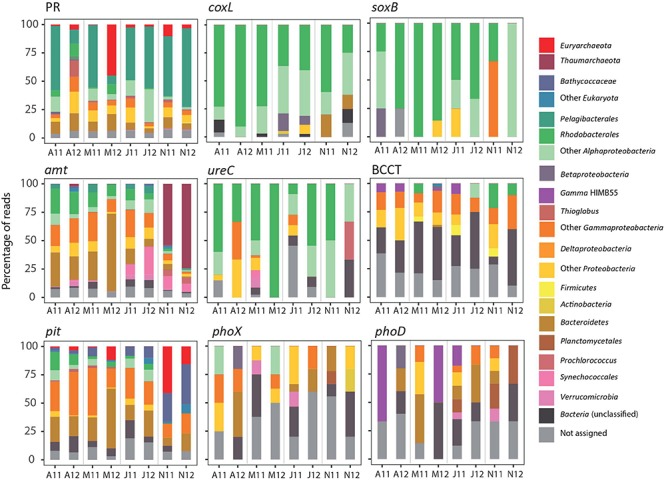
Relative contribution of taxa involved in the expression of each of the marker genes analyzed in the spring (April, May), summer (July) and autumn (November) samples of two consecutive years. Consensus taxonomic bins have been identified using MEGAN software. Only genes which showed active expression in more than 50% of the samples are shown.

Due to our limited sequencing depth, we could not appropriately capture rare transcripts. Yet, the coverage should be enough for a fair representation of frequently transcribed genes from relatively abundant taxa. Most of the main representatives in the metatranscriptomes were dominant members of surface bacterial communities in temperate waters (i.e., *Pelagibacteraceae*, SAR116, *Rhodobacteraceae*, *Flavobacteriaceae*, etc., Supplementary Figure S1). Some of these taxa showed recurrent seasonal dynamics at station E2 (e.g., *Prochlorococcus*, *Rhodobacteraceae*), while the dominant taxa affiliated with *Pelagibacteraceae* did not show any significant seasonality (Alonso-Sáez et al., 2015). A drastic decrease in the abundance of *Pelagibacteraceae* was found in samples collected in spring 2012 (Alonso-Sáez et al., 2015), consistent with the atypical cytometric profiles found in those samples (Morán et al., 2015). This marked change in composition was mirrored by substantial changes in the expression of some marker genes, such as PRs (Figure 2). The PR gene was mainly expressed by *Pelagibacteraceae* and their transcripts showed minimum levels in spring 2012, when its expression was dominated by *Gammaproteobacteria* (including *Thioglobus*), *Bacteroidetes* and *Euryarchaeota* (Figure 3). We also found a significant correlation between PR transcripts and the abundance of SAR11 *in situ* (determined by 16S rRNA amplicon sequencing in Alonso-Sáez et al., 2015, Spearman *Rho* = 0.83, *p* = 0.015, *n* = 8). Similarly, in coastal waters of the San Pedro Channel, PR gene transcripts were generally dominated by SAR11 except for a sample collected during a spring algal bloom, where Gammaproteobacteria dominated PR transcripts (Sieradzki et al., 2018). Thus, our results support the idea that community turnover strongly impacts some gene transcriptional patterns (Salazar et al., 2019). Additionally, the regulation of PR genes is likely complex at the community level, as PR have been found to be constitutively expressed (Giovannoni et al., 2005; Riedel et al., 2010) or regulated in response to light, nutrients or physiologic conditions in different isolates (Gómez-Consarnau et al., 2007, 2016; Steindler et al., 2011; Akram et al., 2013) and in the environment (Lami et al., 2009). Here, we found a marginally significant negative correlation between PR expression levels and cell-specific bacterial production (Spearman *Rho* = −0.71, *n* = 7, *p*-value = 0.088), supporting the view that the expression of PRs may decrease under conditions of high C bioavailability (McCarren et al., 2010).

In comparison to PRs, the expression of the marker gene *pufM*, encoding the M subunit of the Aerobic Anoxygenic Photosynthesis (AAP) reaction-center complex was very low (at least two orders of magnitude below PRs, Figure 2). However, the quantification of AAP activity may have been underestimated in our dataset as samples were always collected around midday and the expression of *puf* genes takes place mostly during the night (Wagner-Döbler and Biebl, 2006; Jeanthon et al., 2011; Voget et al., 2015). We did not recover any *pufM* transcripts in autumn (November), a season when the abundance of AAPs has been described to rapidly drop in Mediterranean waters, likely associated with decreases in temperature and light availability (Ferrera et al., 2014).

With regards to the N cycle, a temporal succession of the key processes nitrogen fixation and ammonia oxidation was suggested by the dynamics of *nifH* and *amoA* genes (Figure 2). In our dataset, most nitrogenase related transcripts (96%) were affiliated with the unicellular symbiotic cyanobacterium *Can.* A. thalassa (previously UCYN-A, Thompson et al., 2012), sharing 93–100% identities at the nucleotide level. A previous study had also detected UCYN-A in summer at a nearby marine site, by using a double Catalyzed Reporter Deposition - Fluorescence *In Situ* Hybridization (CARD-FISH, Cabello et al., 2016). This taxon has been recognized as one of the most abundant nitrogen-fixing organisms in the surface open ocean (Martínez-Pérez et al., 2016), while some studies have shown a more widespread distribution, including deeper water layers and coastal environments (Moisander et al., 2010). As N fixation may shift the communities to P limitation, documenting the relevance of this process has important biogeochemical implications. At the station E2-Gijón/Xixón, evidence for N and P co-limitation of heterotrophic prokaryotes has been suggested for most of 2012 (with nitrate and phosphate concentration below 1 and 0.1 μmoL L^–1^, respectively, Morán et al., 2018). Yet, these conditions were not restricted to the summer months, when we detected *nifH* transcriptional activity, but comprised from May through November. In general, the response of *Can*. A. thalassa to nutrient availability in terms of N fixation and *nifH* transcription has shown mixed responses, with no clear pattern (Turk-Kubo et al., 2012; Krupke et al., 2015). In a recent study, where the abundance of UCYN-A1 was analyzed in surface samples from the global ocean, no environmental factor clearly explained their distribution, but it was suggested that light availability may limit its growth (Cabello et al., 2016). Here, we found evidence that *Can*. A. thalassa were active in summer in mid-shelf waters, while supporting the observations that, in contrast to other unicellular diazotrophic cyanobacteria, they fix N during the day (Muñoz-Marín et al., 2019).

In contrast to the *nifH* expression dynamics, transcripts of another key enzyme of the N cycle was almost only found in autumn: the ammonia monooxygenase *(amo)* involved in archaeal nitrification (Wuchter et al., 2006). Interestingly, recurrent peaks of ammonia-oxidizing archaea have been found in autumn and winter in other coastal systems (Galand et al., 2010; Pitcher et al., 2011). While the ultimate reasons for such seasonal patterns remain unclear, the combination of temperature, nutrient availability, light and more recently, ROS sensitivity (Tolar et al., 2016), have been suggested as possible drivers of their dynamics. Presumably to fuel their nitrifying activity, *Thaumarchaeota* contributed significantly to the uptake of ammonia through the *amt* membrane transporter in autumn (up to 74% of total *amt* transcripts, Figure 3). Similarly, N acquisition proteins were dominant in thaumarchaeal transcripts in Sapelo Island waters (Hollibaugh et al., 2014). The high normalized transcript abundance of *amoA*, as compared to other genes (e.g., *nifH*, *soxB*, *coxL*) indicates a very active transcriptional activity of *Thaumarchaeota*. This is remarkable given their low abundance *in situ* (on average only ca. 1% of cells as determined by CARD-FISH; Alonso-Sáez et al., 2015). These results agree with previous studies where *Thaumarchaeota* were also highly represented at the transcriptional level despite sustaining low-abundant populations (Church et al., 2010; Gifford et al., 2011), and suggests a key role for these microorganisms in the N biogeochemistry also in temperate systems.

While polar archaea can fuel nitrification by using urea (Alonso-Sáez et al., 2012), isolate-based studies have shown that the capability to use N from urea is not universal within *Thaumarchaeota* (Walker et al., 2010; Qin et al., 2014; Bayer et al., 2016). We did not detect transcripts of archaeal ureases at the E2 station, in agreement with the finding that ureases are more abundant in polar than temperate *Thaumarchaeota* (Tolar et al., 2016). However, a diversity of bacterial ureases was actively expressed, consistent with the idea that a large diversity of bacteria can use this compound (Collier et al., 2009; Solomon et al., 2010). Most ureolytic bacteria were affiliated with *Rhodobacterales* and other *Alphaproteobacteria*, but occasional active expression of *Gammaproteobacteria* and cyanobacteria was also found (Figure 3). *Prochlorococcus* was actively expressing *ureC* genes and *amt* transporters in autumn, in line with results from transcriptomic studies of the model strain *Prochlorococcus* sp. MED4, which expressed both mechanisms of N acquisition simultaneously (Zinser et al., 2009). While the relative abundance of *ureC* transcripts was lower than the widespread ammonia transporter *amt*, it is still remarkable that the temporal dynamics of both transcripts was largely similar (Figure 2). This suggests that, from the temporal perspective, both substrates can be simultaneously incorporated by coastal marine bacteria as N sources.

In addition to nitrifiers, some marine microbes can oxidize other widespread substrates as an alternative energy source, such as carbon monoxide (CO) and inorganic S compounds. These processes were targeted by the marker genes encoding the CO dehydrogenase large subunit (*coxL*) and the sulfate thiohydrolase (*soxB*) of the Sox multi-enzyme pathway. *Rhodobacterales* were major contributors to the transcript pool of both *soxB* and *coxL* (Figure 3), confirming the view that members of this clade are central to the pelagic S cycling and CO metabolism (Buchan et al., 2005; Wagner-Döbler and Biebl, 2006). Transcripts of *coxL* and *soxB* genes showed very different dynamics as compared to *amoA*, suggesting a different temporal partitioning of these energy-conservation strategies (Figure 2). The use of the supplementary energy gained by CO oxidation has previously been described as a strategy for coping with nutrient-poor conditions in *Rhodobacteraceae* (Moran et al., 2004), but the energetic benefits of oxidizing CO so far remain unclear (Cunliffe, 2013; Giebel et al., 2019). Here, we found an inverse relationship between *coxL* expression and chlorophyll *a* concentration (Spearman Rho = −0.738, *p* = 0.046, *n* = 8) as a proxy for trophic status, in agreement with the idea that bacteria can use CO to obtain extra energy under substrate limiting conditions.

Related with S metabolism, the expression of a DMSP transporter (BCCT) peaked both years in the phytoplankton decay period, characterized by potentially high availability of DMSP released from microalgae (Yoch, 2002). While most of the transcripts were not confidently assigned to any specific bacterial taxon, *Gammaproteobacteria* contributed ca. 20% of BCCT transporter transcripts, some of them identified as HIMB55, a member of the OM60/NOR5 clade (Figure 3). Previous results using microautoradiography combined with FISH have shown that a taxonomically diverse suite of microbes is potentially involved in DMSP uptake, including some unidentified *Gammaproteobacteria* (Vila et al., 2004; Malmstrom et al., 2005; Motard-Côté et al., 2012). Our results by metatranscriptomics, together with those of Vila-Costa et al. (2010) support this view, and identified the gammaproteobacterium HIMB55 and *Rhodobacterales* as potentially active consumers.

Finally, regarding the phosphorus (P) cycle, a number of mechanisms for the acquisition of this element were analyzed. As P is a key component of cell macromolecules, prokaryotes have developed different strategies to incorporate it, such as the production of extracellular alkaline phosphatases to scavenge Pi from polymers. At least three prokaryotic alkaline phosphatase gene families have been described (*phoA, phoD*, and *phoX*), which differ in substrate specificity and requirements of specific metal ions. The two most highly expressed extracellular phosphatases in marine waters are PhoD and PhoX (Luo et al., 2009; Sebastián and Ammerman, 2009), while the most classical PhoA is mainly intracellular, and likely playing a role in internal organophosphate hydrolysis. We found maximum abundance of both *phoD* and *phoX* transcripts in summer 2011. In contrast, the low-affinity P transporter *pit* clearly showed different dynamics, likely reflecting P availability conditions at the study site (Morán et al., 2018). As found elsewhere (Luo et al., 2009), most *phoD* and *phoX* transcripts remained uncharacterized at the taxonomic level (Figure 3), while *Gammaproteobacteria* and *Bacteroidetes* dominated *pit* transcripts. These groups include copiotrophic taxa with mechanisms of luxury P acquisition to meet their high demands for cellular energetics and growth. Thus, the dynamics of the *pit* transporter is likely associated with the presence of these taxa typically abundant in post-bloom conditions. Interestingly, in autumn, some picoeukaryotes (mainly *Ostreococcus*) and *Euryarchaeota*, an ecologically significant group with yet poorly known metabolic features (Zhang et al., 2015), jointly contributed more than 50% of *pit* transcripts, indicating their relevance in P cycling at that time of the year (Figure 3). Thus, our results indicate a so far unrecognized important role in nutrient cycling for Archaea in temperate waters. Often disregarded because of their low contribution to total prokaryotic abundance in surface waters, they may also represent key players in the biogeochemical cycling of coastal environments.

In summary, we have found evidence of active expression of diagnostic genes of some key microbial processes, such as nitrogen fixation, nitrification and carbon monoxide oxidation at a mid-shelf temperate site, which showed contrasting and occasionally recurrent patterns in their temporal dynamics. This suggests that the associated biogeochemical activities are liable to be affected by seasonally changing environmental conditions. We confirmed a prominent role of *Thaumarchaeota* in ammonia oxidation, *Rhodobacterales* in the use of alternative energy conservation strategies (CO and reduced sulfur oxidation) and obtained new hints into the microbial taxa actively involved in other biogeochemical processes in coastal shelf waters, such as *Can.* A. thalassa in nitrogen fixation, OM60/NOR5 in DMSP uptake, and *Euryarchaeota* and *Ostreococcus* in P cycling.

## Data Availability Statement

Raw sequences analyzed for this study can be found in the European Nucleotide Archive under the following accession numbers: ERS1836494–ERS1836501.

## Author Contributions

LA-S and XM conceived the study. LA-S conducted the sample collection and laboratory processing, and wrote the manuscript with input from all other authors. LA-S and JG analyzed the transcriptomic data.

## Conflict of Interest

The authors declare that the research was conducted in the absence of any commercial or financial relationships that could be construed as a potential conflict of interest.
