# Supplementary material for: Transcriptional Patterns of Biogeochemically Relevant Marker Genes by Temperate Marine Bacteria
Source: Front Microbiol. 2020 Mar 20;11:465. doi: 10.3389/fmicb.2020.00465 (PMC7098952; doi:10.3389/fmicb.2020.00465)
Supplement: Supplementary file 1 [file Data_Sheet_1.PDF]

Supplementary Material

“Transcriptional patterns of biogeochemically relevant marker genes by temperate marine bacteria” by L Alonso-Sáez, XAG Morán, JM González

Supplementary Table S1. Number of reads and average length (in nucleotides) obtained from the Illumina Miseq run. Raw reads, reads after quality trimming and rRNA removal, and significant hits against the Refseq database (version 63, BLASTx, Bit-score > 40) are shown.

	**Raw Reads**		**Quality Trimmed reads**		**Non-rRNA reads**	**Refseq BLASTx hits**
	**Number**	**Length**	**Number**	**Length**	**Number**	**Number**
21 April 2011	1 833 313	237	1 290 799	152	532 706	401 098
22 May 2011	3 728 913	215	2 599 188	151	1 222 358	889 964
14 July 2011	2 614 931	214	1 795 529	148	564 572	323 272
17 Nov 2011	4 197 507	218	2 821 587	149	780 955	409 868
2 May 2012	1 385 140	215	990 603	150	372 780	227 686
23 May 2012	1 094 949	209	784 096	150	201 510	104 247
3 July 2012	1 442 785	219	998 929	148	322 105	189 220
15 Nov 2012	1 535 942	218	1 054 262	150	262 370	146 455
**TOTAL**	**17 833 480**		**12 334 993**		**4 259 356**	**2 691 810**

Supplementary Table S2. Number of hits to relevant functional marker genes obtained by BLASTx of the metatranscriptomes against the Refseq database (version 63, BLASTx bit-score > 40) and subsequent search by text-based query.

Function	Text-query	A11	M11	J11	N11	A12	M12	J12	N12
**Photoheterotrophy**	Rhodopsin	4957	4973	1174	1533	219	244	2489	536
**Photoheterotrophy**	Bacteriochlorophyll synthase	4	4	4	2	3	0	1	2
**N cycle**	Nitrogenase	1	13	138	0	0	1	4	1
**N cycle**	Ammonium transporter	7670	82836	3983	1647	464	713	1283	923
**N cycle**	Ammonia monooxygenase	9	10	4	1328	10	2	0	278
**N cycle**	Nitrate transporter	1	84	82	2	0	2	11	4
**N cycle**	Nitrate reductase	32	42	19	19	4	5	10	9
**N cycle**	Nitrite reductase	15	191	116	197	22	15	10	19
**N cycle**	NirK, nitrite reductase	0	0	1	63	0	1	0	10
**N cycle**	Urease	110	425	158	35	9	10	33	7
**P cycle**	Alkaline phosphatase	43	117	71	49	18	14	25	17
**P cycle**	Phosphate transporter	72	183	41	74	117	30	16	27
**P cycle**	Phospholipase	55	103	57	39	33	25	21	11
**S cycle**	Sulfur oxidation	57	107	38	25	31	1	18	3
**S cycle**	Dimethyl sulfoniopropionate demethylase	53	92	36	27	22	9	16	17
**Methylotrophy**	Serine-glyoxylate aminotransferase	11	71	18	3	1	0	9	1
**Methylotrophy**	Formate dehydrogenase	2213	2822	1110	610	678	196	971	225
**Methylotrophy**	Methanol dehydrogenase	1411	2835	584	371	486	41	573	162
**Methylotrophy**	Methenyltetrahydromethanopterin cyclohydrolase	55	34	19	2	0	26	10	1
**Methylotrophy**	Crotonyl-CoA reductase	7	100	46	18	3	2	7	6

**Methylotrophy**	Formaldehyde-activating enzyme	42	48	12	15	5	13	3	7
**Methylotrophy**	Carbon monoxide dehydrogenase	192	335	344	100	103	23	139	47

**Function**	**Text-query**	**A11**	**M11**	**J11**	**N11**	**A12**	**M12**	**J12**	**N12**
**Oxidative Genes**	Aminopeptidase	482	1110	637	601	355	192	253	143
**Oxidative Genes**	Glucosidase	105	225	75	70	69	66	33	12
**Oxidative Genes**	Glycosidase	18	82	37	14	15	10	5	1
**Oxidative Genes**	Lipase	85	202	152	87	60	43	39	22
**Oxidative Genes**	Sulfatase	267	1262	579	237	97	73	183	68
**Oxidative Genes**	Chitinase	8	9	4	8	4	9	5	4

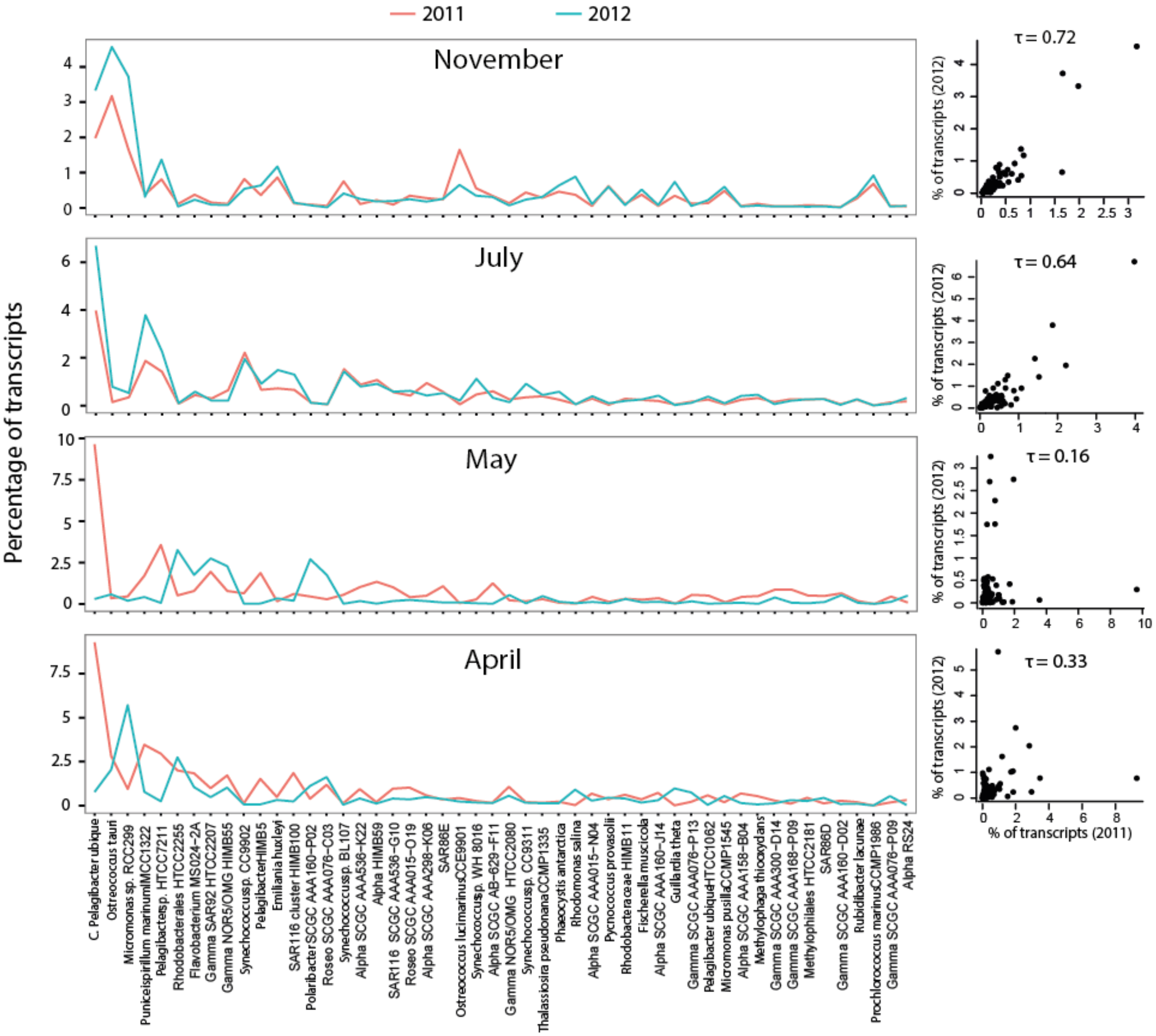

Figure S1. Contribution of the 50-top expressed taxonomic bins to the metatranscriptomes over the two consecutive years analysed. The corresponding correlation analyses appear on the right side and the Kendall *Thau* correlation coefficients are shown ($P < 0.05$).
